# Thoracic and diaphragmatic endometriosis: Single-institution experience using novel, broadened diagnostic criteria

**DOI:** 10.4274/jtgga.2018.0035

**Published:** 2018-08-06

**Authors:** Demetrio Larraín, Francisco Suárez, Hernán Braun, Javier Chapochnick, Lidia Diaz, Iván Rojas

**Affiliations:** 1 Department of Obstetrics and Gynecology, Endometriosis Unit, Clínica Santa María, Santiago, Chile; 2 Department of Thoracic Surgery, Clínica Santa María, Santiago, Chile; 3 Department of Obstetrics and Gynecology, Clínica Santa María, Santiago, Chile; 4 Department of Hepatobiliary Surgery, Clínica Santa María, Santiago, Chile; 5 Department of Pathology, Clínica Santa María, Santiago, Chile

**Keywords:** Thoracic endometriosis syndrome, diaphragmatic endometriosis, thoracic endometriosis

## Abstract

**Objective:**

To describe our experience with the multidisciplinary management of both thoracic/diaphragmatic endometriosis (TED), applying a broadened definition of the “Thoracic endometriosis syndrome (TES)” to define cases.

**Material and Methods:**

We present a retrospective series of consecutive patients affected by pathology-proven TED, treated at our institution, during a period of 7 years.

**Results:**

Five women were included. Two patients were referred due to catamenial chest/shoulder pain, one due to recurrent catamenial pneumothorax, and one due to new-onset diaphragmatic hernia. One patient had no thoracic symptoms, but diaphragmatic endometriosis was found during gynecologic laparoscopy for pelvic endometriosis. Endometriosis was histologically confirmed in all cases. After follow-up, all patients remain asymptomatic.

**Conclusion:**

Broadened TES criteria could increase the incidence of TED and determine better knowledge of this condition. Multidisciplinary, minimally invasive surgery is effective and safe, but should be reserved for tertiary referral centers.

## Introduction

Endometriosis is defined as the presence of endometrial glands and stroma outside the endometrial cavity; it can be categorized as pelvic and extrapelvic, based on anatomic distribution. It has been estimated that about 12% of women with endometriosis have extrapelvic disease, but the exact prevalence of diaphragmatic endometriosis is unknown (1). Endometriotic involvement of the diaphragm is often asymptomatic, but when symptoms occur they consist of chest pain, upper quadrant pain, and catamenial shoulder pain. These symptoms may or may not occur in relation to menstruation (2). Diaphragmatic endometriosis can also present with what is called thoracic endometriosis syndrome (TES), an entity that classically includes catamenial pneumothorax, catamenial hemothorax, catamenial hemoptysis or intrathoracic endometriotic nodules (3). However, a recent study has proposed including another three entities in the spectrum of TES, namely, endometriosis-related diaphragmatic hernia, catamenial chest pain, and endometriosis-related pleural effusion (4). We hypothesize that this “extended” definition of TES could increase the sensitivity of diagnosis of diaphragmatic endometriosis and lead to a better knowledge of this condition. The objective of the present study was to review our single-institution experience with the diagnosis and management of TES and diaphragmatic endometriosis using this recently-proposed criteria.

## Material and Methods

This is a retrospective observational study of all patients with a definitive diagnosis of diaphragmatic or thoracic endometriosis who were managed at our institution between January 2010 and October 2017. In our institution the diagnosis, of TES/diaphragmatic endometriosis can be first established in different ways: First, by thoracic surgeons, either at the time of video-assisted thoracoscopic surgery (VATS) or in patients presenting with TES but without previous diagnosis of endometriosis; these patients are referred to a gynecologic team for further treatment. Second, by the gynecologists, in patients with known history of pelvic endometriosis who present with TES, or as an incidental finding during gynecologic laparoscopy. These patients are either referred to the thoracic surgery team or evaluated by them intraoperatively. We included all patients who were evaluated for clinically-suspected diaphragmatic endometriosis or “extended” TES (i.e., including endometriosis-related diaphragmatic hernia, catamenial chest pain, and endometriosis-related pleural effusion) and those incidentally found to have diaphragmatic endometriosis during gynecologic laparoscopy for endometriosis. We consider this as a valid tool for diagnosis of diaphragmatic endometriosis because we routinely inspect the diaphragm for endometriosis at the time of laparoscopy. Visual diagnosis was confirmed with pathology whenever possible; however, in cases in which thoracic/diaphragmatic samples were not obtained, only cases with pathologic confirmation of endometriosis from other sites were included. Furthermore, we included all patients with a diagnosis of thoracic or diaphragmatic endometriosis in definitive pathology examinations obtained from both gynecologic and thoracic surgery databases. Available data about clinical symptoms, medical treatments, and surgical findings were tabulated and correlated with both clinical and pathologic features for each patient. Institutional Review Board approval was obtained from the Institutional Ethics Committee. Informed consent was obtained from all patients.

## Results

We found five cases of thoracic/diaphragmatic endometriosis during the study period. Three patients were referred to our unit for evaluation from the thoracic surgery department, and the other two were primarily evaluated at our own gynecology department.

### Case 1

**Figure 1:**
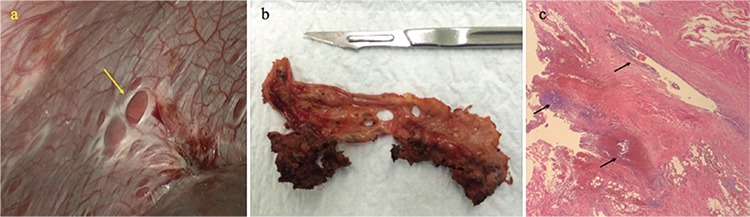
a) Laparoscopic aspect of diaphragmatic endometriosis. Note the “typical” fenestrations on the diaphragmatic surface (arrow). b) Macroscopic view of the surgical specimen in the same patient. c) Microscopic view of endometrial tissue (glands and stroma) in the resected diaphragm (arrows) (Hematoxylin & Eosin stain, ×10)

A 36-year-old woman, gravida 1, para 1, with a history of laparoscopic fulguration of pelvic endometriosis 3 years previously was referred to our institution because of dysmenorrhea and monthly right-sided shoulder pain associated with menses. She was using oral contraceptive pills (OCP) without pain relief. Deep pelvic endometriosis with endometriotic involvement of the diaphragm was suspected and abdominopelvic and diaphragmatic magnetic resonance imaging (MRI) was performed. MRI showed deep pelvic endometriosis involving the bladder and the uterosacral ligaments, and multiple posterior subphrenic lesions suggestive of diaphragmatic endometriosis. We performed a multidisciplinary team laparoscopy (gynecologic and thoracic surgeons) and found deep pelvic endometriosis and extensive endometriosis involving the right posterior hemidiaphragm (only visible with the 30º optic and after liver mobilization). Laparoscopic partial cystectomy, uterosacral ligament resection and a full-thickness partial diaphragmatic resection were performed without complications; a chest drain was left in place for 2 days and she was discharged on postoperative day 5. Histopathology confirmed endometriosis in all specimens (Figure 1a-c). To date, she is using OCP and remains asymptomatic after 38 months’ follow-up.

### Case 2

**Figure 2:**
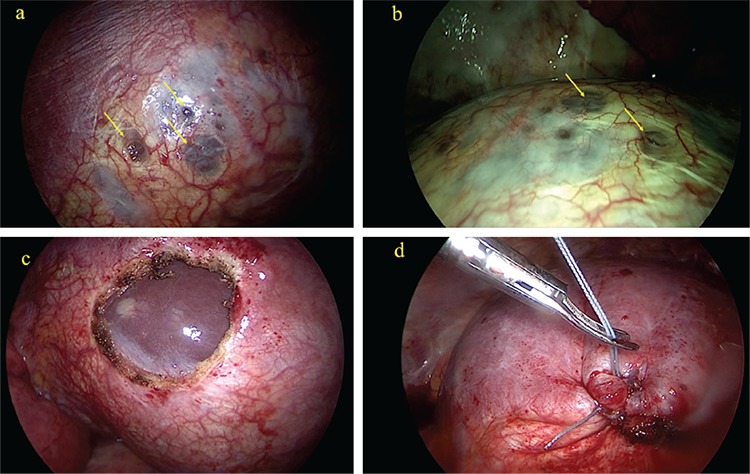
a, b) Thoracoscopic aspect of diaphragmatic endometriosis. Note the presence of fenestrations on the thoracic surface of the diaphragm (arrows). c) Diaphragmatic defect after surgical resection. The liver surface is visible through the defect. d) Diaphragmatic suture after endometriosis resection

A 42-year-old nulliparous woman with a history of infertility and recurrent catamenial pneumothorax (2 previous episodes, the last one 6 months earlier) was referred to our emergency department due to right-sided chest pain and mild dyspnea, which started within 48 hours of onset of menses. She had no previous history of endometriosis and never had dysmenorrhea or dyspareunia. The initial examination included chest X-ray, which revealed a right pneumothorax. A chest computed tomography (CT) scan confirmed the diagnosis and VATS was performed. During VATS, we found several diaphragmatic fenestrations that communicated with the abdominal cavity, through which the liver had herniated. The involved area was resected and the diaphragm was repaired using a nonabsorbable interrupted suture (Figure 2a-c). Pathologic report confirmed diaphragmatic endometriosis. The patient underwent in vitro fertilization (IVF) 4 months after surgery and became pregnant. She is now at 20-weeks of a normal pregnancy and remains asymptomatic.

### Case 3

A nulliparous, 26-year-old woman, with a known diagnosis of pelvic endometriosis and medical treatment with continuous OCP for 9 years was referred to our institution due to recurrent pelvic pain, severe dysmenorrhea, and dyspareunia. Moreover, she presented with chronic right shoulder pain, which was exacerbated during menstruation. She had a history of one previous laparoscopy for endometriosis in which both pelvic and diaphragmatic endometriosis were discovered, but the latter was not treated. The biopsy confirmed endometriosis in all pelvic samples. After the surgery she was treated with gonadotrophin-releasing hormone (GnRH) analogues for six months with transitory improvement, but she could not receive more OCP due to de discovery of a hepatic adenoma. Due to persistent and incapacitating catamenial right shoulder pain accompanied by severe dyspnea, a chest CT was performed with only nonspecific findings. She underwent an exploratory VATS and we found several endometriotic foci in the central tendon of the diaphragm and right hemidiaphragm, which were fulgurated and resected. The pathology report was consistent with fibrosis but not with endometriosis. However, it also reported a marked thermal effect on the tissue. Four months after surgery she conceived spontaneously and delivered a healthy newborn at 38 weeks of gestation. She is currently under treatment with an implantable contraceptive and reports great improvement after a 7-year follow-up.

### Case 4

A 35-year-old nulliparous woman, was referred to our unit with a long history of infertility and chronic pelvic pain. She also had severe dysmenorrhea and dyspareunia, but reported no thoracic symptoms. She underwent gynecologic laparoscopy, and deep pelvic endometriosis in the uterosacral ligaments was resected; several endometriotic lesions in the right hemidiaphragm were left behind and not treated, due the lack of symptoms. The pathology report confirmed endometriosis in all pelvic specimens. Fourteen months after surgery, she underwent three cycles of intrauterine insemination and became pregnant. She underwent emergency cesarean section at 30 weeks of gestation due to placental abruption with good perinatal outcome. She remains asymptomatic after 55 months’ follow-up.

### Case 5

A 40-year-old nulliparous woman, with history of four previous surgeries for endometriosis, persistent dysmenorrhea, and infertility was evaluated in our emergency department due to epigastric and left flank pain, dyspepsia, and nausea. Abdominopelvic CT revealed a left diaphragmatic hernia, with the splenic flexure of the colon herniated into the chest and signs of severe pelvic endometriosis. Chest CT confirmed the diagnosis and the absence of pneumothorax. She had no history of diaphragmatic surgery, trauma or any pulmonary disease. Moreover, she had undergone chest CT one year earlier due to a deep venous thrombosis, which revealed no diaphragmatic defects. VATS was performed; the edges of the diaphragmatic hernia were resected and the diaphragm was repaired using a direct suture. A pathologic examination of the resected tissue confirmed endometriosis. She remains asymptomatic after 26 months’ follow-up.

## Discussion

**Figure 3:**
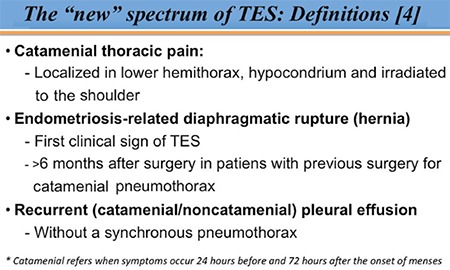
Recently proposed new entities of TES (4) *TES: Thoracic endometriosis syndrome*

TES refers to a broad spectrum of clinical manifestations related to the presence of ectopic endometriotic tissue in the diaphragm or in the thoracic cavity. Despite the many publications on this topic (3,5), only a few include endometriosis-related diaphragmatic hernia, catamenial chest pain, and endometriosis-related pleural effusion as part of TES (4). In the present study, we included these three entities in the TES definition and coined the term “extended TES” (Figure 3). Among our patients, if the “classic” definition was used, we could only include one case of diaphragmatic/thoracic endometriosis. However, when the broadened definition was used in addition to the laparoscopic findings among patients with endometriosis, we identified 5 patients, all of whom had pathologiy-confirmed endometriosis. Hence, rising the incidence in the same population. It has been considered that thoracic/diaphragmatic endometriosis is underdiagnosed and its incidence is often underestimated. It is often overlooked by gynecologists because of the lack of appreciation of the symptoms and the failure to properly examine the patient and evaluate the diaphragm during surgery. In our experience, we routinely inspect the diaphragm for endometriosis during surgery; however, diaphragmatic lesions can easily be missed because they are often located in the posterior diaphragm and hidden behind the liver (6). Furthermore, a significant number of patients with diaphragmatic endometriosis can go undiagnosed for long periods of time because of a traditional focus on the lower pelvic region and the variable appearance of endometriotic lesions (7). Moreover, diaphragmatic endometriosis is usually not systematically associated with pelvic endometriosis (5,8,9). For that reason, some cases are misdiagnosed as other conditions involving the gastrointestinal tract or of cardiothoracic origin. All these factors contribute to a significant delay in the diagnosis (10). In fact, the diagnosis of diaphragmatic endometriosis is often made later in life than that of pelvic disease (3), probably because a longer period of time is needed to affect the diaphragm and thorax. In our series as in others (5,7,10), the median age at diagnosis was more than 35 years. The diagnosis of diaphragmatic endometriosis requires a high level of clinical suspicion, and imaging studies such as chest CT and MRI can assist with diagnosis and surgical planning (11), but they are not mandatory (10).

The pathophysiology of diaphragmatic endometriosis is unknown, however the marked asymmetry in distribution of diaphragmatic and thoracic lesions (90% on the right side), supports the Sampson’s retrograde menstruation theory (12). The most widely accepted mechanism is that endometrial cells in the peritoneal fluid may follow the clockwise peritoneal circulation, through the right paracolic gutter towards the right sub-diaphragmatic area; the phrenicocolic ligament on the left-hand side and the falciform ligament form barriers that prevent cells and fluid from reaching the left sub-diaphragmatic area (12). However, lesions have been found on both sides of the diaphragm (2). Endometrial cell implantation leads to the formation of endometriotic nodules on the abdominal side of the diaphragm. The nodules undergo cyclical necrosis and cause diaphragmatic fragility, leading to the formation of the “typical” diaphragmatic fenestrations (Figure 1a). These perforations can coalesce into larger defects that can determine hernia formation. Moreover, it has been reported that diaphragmatic hernia can be the first clinical manifestation of TES (4,13). After endometrial tissue has entered the pleural space, it may colonize other part of the diaphragm or the pleural space. We speculate that the clinical manifestations (i.e., catamenial chest pain, endometriosis-related pleural effusion, endometriosis-related diaphragmatic hernia, catamenial pneumothorax, catamenial hemothorax, intrathoracic endometriotic nodules and catamenial hemoptysis) could be considered as a sequence of events, a consequence of endometrial tissue undergoing cyclical changes. Interestingly, despite diaphragmatic hernia being known as complication in patients with previous surgery for catamenial pneumothorax (10), hernia formation was reported as part of the evolution of diaphragmatic endometriosis when it was diagnosed more than six months after surgery for catamenial pneumothorax (4,14). In that sense, we think that this broadened definition of TES allows both gynecologic and thoracic surgeons a better understanding of the pathophysiology of TES.

Although the definitive diagnosis of endometriosis requires histologic confirmation, it is not always possible due to technical difficulties and the risk of diaphragmatic perforation (2,6), especially in asymptomatic patients. Moreover, the histologic diagnosis of endometriosis may be challenging on small pleural or lung biopsies and it is often overlooked due to the presence of fibrosis, inflammation, the effect of thermocoagulation, and the scant and patchy distribution of endometrial elements (15). This was the situation in Case 3, in which the surgical specimen had a marked thermal effect due to extensive fulguration. Notably, in our experience, in all cases in which diaphragmatic endometriosis was an incidental finding or it was not treated, the disease was confirmed histologically in other sites of the pelvis or abdomen.

In our practice, medical treatment is the first-line approach in patients who have no desire for pregnancy and includes the use of OCP or GnRH analogues. Surgery is reserved for symptomatic patients. We usually start with gynecologic laparoscopy, whereas VATS is reserved for symptomatic patients only who meet the “extended” TES criteria, when nonsurgical treatments have failed.

Surgical approach to diaphragmatic endometriosis has experienced several changes during the last 10 years, from the classic laparotomy/thoracotomy (6), to a less invasive laparoscopy/VATS (1,10,16). In our experience, the diaphragm is routinely inspected during all gynecologic laparoscopies. If better visualization of the diaphragm is required, the patient is positioned in steep reverse-Trendelenburg position and a 30º optic is used. When diaphragmatic lesions are not accessible from suprapubic trocars, additional 5-mm trocars are placed in the upper right or left abdominal quadrant, according to the implant location (16). In some patients (Case 1), liver mobilization is warranted in order to improve exposure (16). If pneumothorax occurred after excision of a full-thickness diaphragmatic nodule, the anesthesiologist is always notified; the resection is completed, and the thoracic surgeon repairs the diaphragmatic defect. However, if diaphragmatic resection is anticipated, a thoracic surgeon is always present in the operative room. VATS allows direct visualization of thoracic implants and nodules, and the ability to resect both parenchymal and diaphragmatic implants (4,10). When VATS is performed as the first procedure, the presence of pelvic endometriosis must be investigated. In our opinion, the best strategy is to perform a multidisciplinary procedure combining laparoscopy and VATS when necessary (1,10).

The necessity for postoperative medical therapy is defined by the gynecologist, and must be individually tailored taking to account patient age, severity of symptoms (pelvic or thoracic), and the desire for pregnancy (17).

Thoracic endometriosis was thought to be an unusual manifestation of extrapelvic endometriosis, but it seems to be increasing, likely because of an increased awareness of the condition among gynecologists and the use of a lower threshold of symptoms to make the diagnosis, such as the “extended” TES criteria. In addition, thoracic endometriosis is often treated as a respiratory disease, reducing opportunities for gynecologic evaluation. Based on our experience, we strongly suggest that patients with cyclic thoracic symptoms and pelvic pain should undergo an evaluation for both thoracic and pelvic endometriosis. For symptomatic patients, the multidisciplinary and minimally invasive surgical approach is safe and reproducible, but it should be reserved for tertiary referral centers where the collaboration between gynecologist and thoracic surgeons is really possible.

The diagnosis of diaphragmatic endometriosis is challenging and requires a high level of clinical suspicion. The use of more sensitive diagnostic criteria could increase the incidence of the disease by including new entities in the classic spectrum of TES. Multidisciplinary evaluation should be the standard in suspected cases. The use of minimally invasive surgical approach is effective and safe, but should be reserved to tertiary referral centers.
